# {2,2′-[Pyridine-3,4-diylbis(nitrilo­methyl­idyne)]diphenolato}zinc(II)

**DOI:** 10.1107/S1600536809038616

**Published:** 2009-10-03

**Authors:** Ning Sheng

**Affiliations:** a Department of Chemistry & Chemical Engineering, Jining University, Qufu 273155, People’s Republic of China

## Abstract

The title compound, [Zn(C_19_H_13_N_3_O_2_)], has been synthesized by the reaction of Zn(ClO_4_)_2_·6H_2_O and the tetra­dentate Schiff base ligand 2,2′-[pyridine-3,4-diylbis(nitrilo­methyl­idyne)]diphenol (*L*). The coordination geometry of the Zn^II^ ion is slightly distorted square-planar, formed by two N atoms and two O atoms from the *L* ligand.

## Related literature

For properties of transition metals complexes with Schiff base ligands, see: Aurangzeb *et al.* (1994 ▶); Hulme *et al.* (1997 ▶); Li *et al.* (2008 ▶); Fei & Fang (2008 ▶); Zhang & Janiak (2001 ▶). For related structures, see: Li & Zhang (2004 ▶); Chen (2005 ▶).
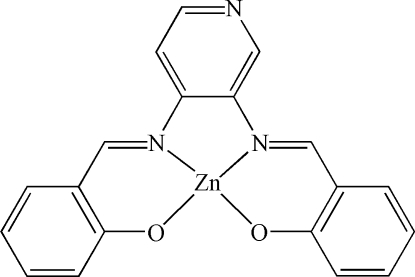


## Experimental

### 

#### Crystal data

[Zn(C_19_H_13_N_3_O_2_)]
*M* *_r_* = 380.69
Orthorhombic, 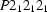

*a* = 5.3563 (8) Å
*b* = 16.603 (2) Å
*c* = 17.311 (3) Å
*V* = 1539.5 (4) Å^3^
*Z* = 4
Mo *K*α radiation
μ = 1.61 mm^−1^
*T* = 293 K
0.25 × 0.21 × 0.18 mm

#### Data collection

Bruker APEXII CCD area-detector diffractometer
Absorption correction: multi-scan (*SADABS*; Sheldrick, 2003 ▶) *T*_min_ = 0.689, *T*_max_ = 0.760
7573 measured reflections
2720 independent reflections
2519 reflections with *I* > 2σ(*I*)
*R*_int_ = 0.032

#### Refinement

*R*[*F* ^2^ > 2σ(*F* ^2^)] = 0.040
*wR*(*F* ^2^) = 0.109
*S* = 1.00
2720 reflections
227 parameters
H-atom parameters constrained
Δρ_max_ = 0.46 e Å^−3^
Δρ_min_ = −0.25 e Å^−3^
Absolute structure: Flack (1983 ▶), 1105 Friedel pairs
Flack parameter: 0.090 (18)

### 

Data collection: *APEX2* (Bruker, 2004 ▶); cell refinement: *SAINT-Plus* (Bruker, 2001 ▶); data reduction: *SAINT-Plus*; program(s) used to solve structure: *SHELXS97* (Sheldrick, 2008 ▶); program(s) used to refine structure: *SHELXL97* (Sheldrick, 2008 ▶); molecular graphics: *XP* (Sheldrick, 1998 ▶); software used to prepare material for publication: *XP*.

## Supplementary Material

Crystal structure: contains datablocks global, I. DOI: 10.1107/S1600536809038616/hg2565sup1.cif

Structure factors: contains datablocks I. DOI: 10.1107/S1600536809038616/hg2565Isup2.hkl

Additional supplementary materials:  crystallographic information; 3D view; checkCIF report

## Figures and Tables

**Fig. 1.**
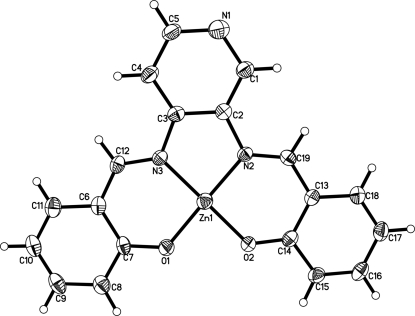
View of the title compound with the atom-labelling scheme. Displacement ellipsoids are drawn at the 30% probability level.

## supplementary crystallographic information

### Comment

Schiff base complexes have attracted much attention due to their interesting structures and wide potential applications. They play an important role in the development of coordination chemistry as well as inorganic biochemistry, catalysis and optical materials (Aurangzeb *et al.*, 1994, Hulme *et al.*, 1997; Li *et al.*, 2008; Fei *et al.*, 2008; Zhang & Janiak, 2001). Here, we report the structure of a new zinc complex based on a tetradentate Schiff base ligand. The molecular structure of title compound is shown in Fig. 1. As can be seen, the whole molecule of the title complex is essentially planar. The Zn ion is four-coordinate with the four positions  occupied by two N atoms and two O atoms of the Schiff base ligand. The mean deviation of the plane formed by ZnN_2_O_2_ unit is 0.0121 Å. The Zn—O and Zn—N bond lengths are all consistent with those found in other Zn Schiff base complexes (Chen, 2005; Li, *et al.*, 2004).

### Experimental

The Schiff base ligand was synthesized by condensation 3,4-diaminopyridine and 2-hydroxy-benzaldehyde with the ratio 1:2 in ethanol.The synthesis of the title complex was carried out by reacting Zn(ClO_4_)_2_.6H_2_O (1 mmol, 373 mg) and the schiff-base ligand (1 mmol, 317 mg) in methanol under the stirring condition at room temperature. The filtrated solution was left to slowly evaperate in air to obtain single-crystal suitable for X-ray diffraction with the yield about 228 mg, 60%.

### Refinement

All the H atoms bonded to the C atoms were placed using the HFIX commands in *SHELXL-97*, with C—H distances of 0.93 Å, and were allowed for as riding atoms with *U*_iso_(H) = 1.2*U*_eq_(C).

### Crystal data

**Table:**

[Zn(C_19_H_13_N_3_O_2_)]	*F*(000) = 776
*M**_r_* = 380.69	*D*_x_ = 1.643 Mg m^−^^3^
Orthorhombic, *P*2_1_2_1_2_1_	Mo *K*α radiation, λ = 0.71073 Å
Hall symbol: P 2ac 2ab	Cell parameters from 4002 reflections
*a* = 5.3563 (8) Å	θ = 2.5–26.4°
*b* = 16.603 (2) Å	µ = 1.61 mm^−^^1^
*c* = 17.311 (3) Å	*T* = 293 K
*V* = 1539.5 (4) Å^3^	Block, colourless
*Z* = 4	0.25 × 0.21 × 0.18 mm

### Data collection

**Table:**

Bruker APEXII CCD area-detector diffractometer	2720 independent reflections
Radiation source: fine-focus sealed tube	2519 reflections with *I* > 2σ(*I*)
graphite	*R*_int_ = 0.032
φ and ω scans	θ_max_ = 25.0°, θ_min_ = 1.7°
Absorption correction: multi-scan (*SADABS*; Sheldrick, 2003)	*h* = −6→6
*T*_min_ = 0.689, *T*_max_ = 0.760	*k* = −15→19
7573 measured reflections	*l* = −19→20

### Refinement

**Table:**

Refinement on *F*^2^	Secondary atom site location: difference Fourier map
Least-squares matrix: full	Hydrogen site location: inferred from neighbouring sites
*R*[*F*^2^ > 2σ(*F*^2^)] = 0.040	H-atom parameters constrained
*wR*(*F*^2^) = 0.109	*w* = 1/[σ^2^(*F*_o_^2^) + (0.084*P*)^2^ + 0.0681*P*] where *P* = (*F*_o_^2^ + 2*F*_c_^2^)/3
*S* = 1.00	(Δ/σ)_max_ = 0.001
2720 reflections	Δρ_max_ = 0.46 e Å^−^^3^
227 parameters	Δρ_min_ = −0.25 e Å^−^^3^
0 restraints	Absolute structure: Flack (1983), 1105 Friedel pairs
Primary atom site location: structure-invariant direct methods	Flack parameter: 0.090 (18)

### Special details

**Table:**

Geometry. All esds (except the esd in the dihedral angle between two l.s. planes) are estimated using the full covariance matrix. The cell esds are taken into account individually in the estimation of esds in distances, angles and torsion angles; correlations between esds in cell parameters are only used when they are defined by crystal symmetry. An approximate (isotropic) treatment of cell esds is used for estimating esds involving l.s. planes.
Refinement. Refinement of F^2^ against ALL reflections. The weighted R-factor wR and goodness of fit S are based on F^2^, conventional R-factors R are based on F, with F set to zero for negative F^2^. The threshold expression of F^2^ > 2sigma(F^2^) is used only for calculating R-factors(gt) etc. and is not relevant to the choice of reflections for refinement. R-factors based on F^2^ are statistically about twice as large as those based on F, and R- factors based on ALL data will be even larger.

### Fractional atomic coordinates and isotropic or equivalent isotropic displacement parameters (Å^2^)

**Table:**

	*x*	*y*	*z*	*U*_iso_*/*U*_eq_	
Zn1	0.85000 (8)	0.22279 (3)	0.81022 (2)	0.04025 (17)	
O1	0.5747 (5)	0.18278 (15)	0.76293 (15)	0.0423 (6)	
O2	0.8521 (6)	0.12568 (16)	0.86177 (15)	0.0439 (6)	
N1	1.4213 (8)	0.4650 (2)	0.8360 (2)	0.0592 (10)	
N2	1.1291 (6)	0.26236 (16)	0.85990 (15)	0.0331 (6)	
N3	0.8496 (6)	0.31937 (17)	0.75595 (15)	0.0339 (6)	
C1	1.3829 (8)	0.3878 (2)	0.8649 (2)	0.0455 (9)	
H1	1.4879	0.3673	0.9029	0.055*	
C2	1.1895 (7)	0.3421 (2)	0.83702 (19)	0.0349 (8)	
C3	1.0329 (8)	0.3735 (2)	0.7803 (2)	0.0376 (8)	
C4	1.0658 (9)	0.4516 (2)	0.7538 (2)	0.0474 (10)	
H4	0.9551	0.4738	0.7184	0.057*	
C5	1.2653 (10)	0.4959 (3)	0.7810 (3)	0.0515 (11)	
H5	1.2939	0.5473	0.7617	0.062*	
C6	0.5160 (7)	0.2875 (2)	0.66888 (19)	0.0390 (8)	
C7	0.4547 (7)	0.2126 (2)	0.70379 (19)	0.0374 (8)	
C8	0.2526 (8)	0.1694 (3)	0.6747 (2)	0.0485 (10)	
H8	0.2042	0.1212	0.6976	0.058*	
C9	0.1245 (9)	0.1989 (3)	0.6112 (2)	0.0531 (11)	
H9	−0.0076	0.1689	0.5914	0.064*	
C10	0.1841 (8)	0.2700 (3)	0.5766 (2)	0.0529 (11)	
H10	0.0936	0.2880	0.5341	0.064*	
C11	0.3765 (9)	0.3141 (3)	0.6046 (2)	0.0512 (10)	
H11	0.4173	0.3627	0.5811	0.061*	
C12	0.7065 (7)	0.3362 (2)	0.6972 (2)	0.0385 (8)	
H12	0.7342	0.3849	0.6721	0.046*	
C13	1.2240 (7)	0.1422 (2)	0.9337 (2)	0.0374 (8)	
C14	1.0142 (8)	0.0984 (2)	0.9087 (2)	0.0383 (8)	
C15	0.9911 (8)	0.0187 (2)	0.9372 (2)	0.0451 (9)	
H15	0.8543	−0.0119	0.9220	0.054*	
C16	1.1607 (9)	−0.0145 (3)	0.9858 (2)	0.0492 (10)	
H16	1.1398	−0.0671	1.0029	0.059*	
C17	1.3639 (9)	0.0297 (3)	1.0097 (2)	0.0518 (10)	
H17	1.4790	0.0073	1.0438	0.062*	
C18	1.3951 (8)	0.1056 (2)	0.9835 (2)	0.0453 (10)	
H18	1.5350	0.1346	0.9992	0.054*	
C19	1.2673 (7)	0.2216 (3)	0.9071 (2)	0.0385 (8)	
H19	1.4100	0.2471	0.9255	0.046*	

### Atomic displacement parameters (Å^2^)

**Table:**

	*U*^11^	*U*^22^	*U*^33^	*U*^12^	*U*^13^	*U*^23^
Zn1	0.0432 (3)	0.0352 (2)	0.0423 (3)	0.00271 (19)	−0.0008 (2)	−0.00027 (18)
O1	0.0449 (16)	0.0337 (13)	0.0484 (14)	0.0016 (12)	−0.0065 (12)	0.0014 (11)
O2	0.0449 (15)	0.0393 (13)	0.0475 (14)	−0.0069 (14)	−0.0108 (14)	0.0101 (12)
N1	0.064 (3)	0.055 (2)	0.058 (2)	−0.0059 (19)	0.0026 (18)	−0.0003 (18)
N2	0.0392 (16)	0.0273 (14)	0.0327 (13)	0.0004 (13)	0.0021 (13)	−0.0018 (12)
N3	0.0388 (17)	0.0285 (14)	0.0344 (14)	0.0033 (14)	0.0027 (15)	−0.0011 (11)
C1	0.053 (2)	0.042 (2)	0.0421 (19)	−0.0040 (18)	0.0015 (18)	0.0017 (17)
C2	0.038 (2)	0.0353 (18)	0.0318 (16)	0.0032 (15)	0.0064 (14)	−0.0035 (14)
C3	0.044 (2)	0.0340 (19)	0.0344 (17)	−0.0007 (17)	0.0039 (16)	−0.0040 (14)
C4	0.063 (3)	0.033 (2)	0.046 (2)	0.0008 (18)	−0.002 (2)	0.0048 (17)
C5	0.068 (3)	0.037 (2)	0.050 (2)	−0.0088 (19)	−0.002 (2)	0.0078 (18)
C6	0.042 (2)	0.042 (2)	0.0327 (16)	0.0114 (17)	0.0012 (15)	−0.0041 (15)
C7	0.0357 (17)	0.040 (2)	0.0366 (18)	0.0141 (16)	−0.0035 (15)	−0.0083 (16)
C8	0.046 (2)	0.043 (2)	0.056 (2)	0.0089 (18)	−0.0022 (19)	−0.008 (2)
C9	0.041 (2)	0.062 (3)	0.056 (2)	0.010 (2)	−0.011 (2)	−0.023 (2)
C10	0.047 (2)	0.067 (3)	0.044 (2)	0.014 (2)	−0.0108 (18)	−0.009 (2)
C11	0.054 (3)	0.057 (2)	0.043 (2)	0.011 (2)	0.001 (2)	0.0016 (18)
C12	0.049 (2)	0.0336 (18)	0.0334 (17)	0.0095 (15)	0.0007 (16)	−0.0012 (15)
C13	0.040 (2)	0.038 (2)	0.0343 (17)	0.0016 (16)	0.0017 (16)	0.0028 (15)
C14	0.045 (2)	0.0354 (19)	0.0344 (17)	0.0032 (16)	0.0055 (17)	0.0012 (15)
C15	0.055 (2)	0.0344 (18)	0.046 (2)	−0.0066 (18)	−0.003 (2)	0.0022 (17)
C16	0.060 (3)	0.038 (2)	0.049 (2)	0.011 (2)	0.006 (2)	0.0115 (17)
C17	0.052 (3)	0.053 (2)	0.051 (2)	0.010 (2)	−0.007 (2)	0.0112 (19)
C18	0.046 (3)	0.047 (2)	0.043 (2)	0.0036 (18)	−0.0051 (18)	0.0025 (17)
C19	0.0367 (18)	0.0397 (19)	0.0392 (17)	−0.0019 (17)	−0.0026 (14)	−0.0055 (18)

### Geometric parameters (Å, °)

**Table:**

Zn1—O1	1.813 (3)	C7—C8	1.393 (6)
Zn1—O2	1.843 (2)	C8—C9	1.385 (6)
Zn1—N2	1.846 (3)	C8—H8	0.9300
Zn1—N3	1.858 (3)	C9—C10	1.361 (7)
O1—C7	1.306 (4)	C9—H9	0.9300
O2—C14	1.273 (5)	C10—C11	1.354 (7)
N1—C5	1.367 (6)	C10—H10	0.9300
N1—C1	1.391 (6)	C11—H11	0.9300
N2—C19	1.294 (5)	C12—H12	0.9300
N2—C2	1.419 (5)	C13—C18	1.397 (5)
N3—C12	1.304 (4)	C13—C14	1.406 (6)
N3—C3	1.396 (5)	C13—C19	1.416 (6)
C1—C2	1.372 (6)	C14—C15	1.417 (5)
C1—H1	0.9300	C15—C16	1.355 (6)
C2—C3	1.393 (5)	C15—H15	0.9300
C3—C4	1.387 (5)	C16—C17	1.376 (6)
C4—C5	1.380 (7)	C16—H16	0.9300
C4—H4	0.9300	C17—C18	1.351 (6)
C5—H5	0.9300	C17—H17	0.9300
C6—C12	1.391 (6)	C18—H18	0.9300
C6—C11	1.412 (5)	C19—H19	0.9300
C6—C7	1.421 (6)		
			
O1—Zn1—O2	84.43 (12)	C9—C8—C7	119.4 (4)
O1—Zn1—N2	178.96 (12)	C9—C8—H8	120.3
O2—Zn1—N2	94.64 (12)	C7—C8—H8	120.3
O1—Zn1—N3	94.99 (13)	C10—C9—C8	122.6 (4)
O2—Zn1—N3	178.58 (12)	C10—C9—H9	118.7
N2—Zn1—N3	85.95 (13)	C8—C9—H9	118.7
C7—O1—Zn1	128.0 (3)	C11—C10—C9	119.3 (4)
C14—O2—Zn1	128.6 (2)	C11—C10—H10	120.3
C5—N1—C1	120.4 (4)	C9—C10—H10	120.3
C19—N2—C2	122.3 (3)	C10—C11—C6	121.0 (4)
C19—N2—Zn1	124.9 (3)	C10—C11—H11	119.5
C2—N2—Zn1	112.7 (2)	C6—C11—H11	119.5
C12—N3—C3	120.7 (3)	N3—C12—C6	125.6 (3)
C12—N3—Zn1	125.4 (3)	N3—C12—H12	117.2
C3—N3—Zn1	113.7 (2)	C6—C12—H12	117.2
C2—C1—N1	119.7 (4)	C18—C13—C14	119.4 (3)
C2—C1—H1	120.2	C18—C13—C19	119.8 (4)
N1—C1—H1	120.2	C14—C13—C19	120.8 (3)
C1—C2—C3	119.7 (4)	O2—C14—C13	123.9 (3)
C1—C2—N2	126.2 (4)	O2—C14—C15	119.7 (4)
C3—C2—N2	114.1 (3)	C13—C14—C15	116.4 (3)
C4—C3—C2	120.4 (4)	C16—C15—C14	122.5 (4)
C4—C3—N3	126.3 (4)	C16—C15—H15	118.7
C2—C3—N3	113.3 (3)	C14—C15—H15	118.7
C5—C4—C3	118.9 (4)	C15—C16—C17	120.0 (4)
C5—C4—H4	120.5	C15—C16—H16	120.0
C3—C4—H4	120.5	C17—C16—H16	120.0
N1—C5—C4	120.8 (4)	C18—C17—C16	119.6 (4)
N1—C5—H5	119.6	C18—C17—H17	120.2
C4—C5—H5	119.6	C16—C17—H17	120.2
C12—C6—C11	119.0 (4)	C17—C18—C13	122.1 (4)
C12—C6—C7	121.9 (3)	C17—C18—H18	119.0
C11—C6—C7	119.2 (4)	C13—C18—H18	119.0
O1—C7—C8	118.1 (4)	N2—C19—C13	126.8 (4)
O1—C7—C6	123.5 (3)	N2—C19—H19	116.6
C8—C7—C6	118.4 (3)	C13—C19—H19	116.6
